# Factors associated with survival of ICU patients with pneumonia caused by multidrug-resistant Gram-negative bacteria

**DOI:** 10.1186/cc14190

**Published:** 2015-03-16

**Authors:** M Georgiadou, E Pappa, E Papandreou, H Pavlou, M Eforakopoulou

**Affiliations:** 1 KAT-EKA General Hospital Kifisia, Athens, Greece

## Introduction

Multidrug-resistant (MDR) bacterial pneumonia is associated with significant morbidity and mortality in severely ill ICU patients. The assessment of factors associated with the onset and clinical course of MDR pneumonia may improve treatment effectiveness. The purpose of this study is to identify factors associated with outcome in mechanically ventilated patients with ventilator-associated pneumonia (VAP) caused by MDR bacteria.

## Methods

We studied retrospectively all mechanically ventilated patients treated in the A' ICU of KAT General Hospital in Athens from 1 January 2011 to 31 December 2013 and showed ventilator-associated pneumonia from MDR Gram-negative bacteria. Standard demographic and clinical data, the causative organisms and outcome were recorded. For statistical significance, chi-square and Student *t *tests were used.

## Results

A total of 102 mechanically ventilated patients, 75 men and 27 women, were included in the study. All patients showed VAP caused by MDR bacteria. They were stratified by outcome into survivors and nonsurvivors. ICU mortality was 55%. Gender, cause of admission, the causative microbe, colonization of bronchial secretions and secondary bacteremia had no correlation with outcome. Age and APACHE II score were higher in nonsurvivors (*P *< 0.01 and *P *< 0.05 respectively). The time-onset of pneumonia after admission was longer in patients with VAP caused by Klebsiella or Pseudomonas than those with VAP caused by acinetobacter (*P *< 0.01). Patients with Klebsiella or Pseudomonas pneumoniae needed more time on mechanical ventilation than those with pneumonia from acinetobacter (*P *< 0.01).

## Conclusion

VAP caused by MDR bacteria is a leading cause of ICU death. Age and APACHE II score are significant risk factors of death.
